# Sucrose fuels liver cancer cell growth and spheroid formation: New insights from 2D and 3D cell culture models

**DOI:** 10.3892/mi.2026.311

**Published:** 2026-03-24

**Authors:** Gamze Demirel, Cagdas Kaya, Ranan Gulhan Aktas

**Affiliations:** 1 Department of Medical Biology and Genetics, Faculty of Medicine, Maltepe University, 34857 Istanbul, Türkiye; 2 Department of Biotechnology, Istanbul University Institute of Science, 34452 Istanbul, Türkiye; 3 Istanbul Training and Research Hospital, Department of Internal Medicine, 34080 Istanbul, Türkiye; 4 Cellorama, Milton, MA 02186, USA

**Keywords:** 3D cell culture, alpha-fetoprotein, albumin, cell membrane, spheroids, sucrose

## Abstract

Although there have been studies on the effect of sucrose on cell growth in various cancer types and spheroid formation, further research on whether sucrose supports the development of liver cancer cells would be instrumental for the development of more effective *in vitro* 3D models for drug discovery. Thus, the present study examined the effects of sucrose on the growth of liver cancer cells. The results revealed that sucrose at a concentration of 10 mM/l was associated with the increased growth of liver cancer cells and enhanced their ability to form spheroids. These findings challenge previous assumptions, as spheroid formation is associated with increased tumor aggressiveness. Furthermore, the observed increase in the levels of in alpha-fetoprotein (a marker for liver cancer) with unaltered albumin levels (a marker for healthy liver function) suggested a potential shift towards a more malignant state. Live imaging revealed no significant differences in cell membrane behavior and the morphology of the cancer cells between the sucrose-treated and untreated groups. On the whole, while the present study primarily focuses on phenotypic outcomes, the mechanisms underlying these observations remain to be elucidated in future studies.

## Introduction

Regarded as a primary cause of mortality in cirrhosis, liver cancer ranks fourth globally in terms of cancer-related mortality. With fatalities roughly matching global incidence rates, the prognosis for hepatocellular carcinoma (HCC) is poor (1). HCC is the fifth most common liver cancer among men and the seventh most common type of cancer among women, accounting for 9.2% of global cancer cases (2). The major risk factor associated with HCC is chronic liver injury resulting from any etiology that progresses to cirrhosis. Globally, hepatitis B virus and hepatitis C virus infection are the main causal agents of cirrhosis, and the incidence rate of HCC development in patients with established cirrhosis is ~2 to 4% per year (1,2). Anatomic stage, biologic grade and severity of cirrhosis, determine the survival of patients with HCC (3).

One characteristic that sets HCC apart is the survival advantage of the cancer cells. The liver is constantly adapting to changes in the external environment caused by viruses, dietary xenobiotics and microbiota agents (4). The unbounded lifespan, stable phenotype, great availability and the ease of handling of hepatoma cell lines set them apart. Their primary drawback, in contrast to hepatocytes, is the reduced expression of certain metabolic activities. As human liver cancer cells derived from a patient, HepG2, are the most frequently used cell line in hepatotoxicity and drug metabolism studies. These cells have a chromosomal count of 55, an epithelial display appearance, and carry out a variety of distinct liver activities (5).

Monolayer cell culture, which is a more prevalent culture method where cells typically grow in a flat plane on a glass or polystyrene petri dish, has become a traditional approach. However, the increasingly expansive field of 3D cell culture involves cells growing in a third dimension, often in spherical or spheroid structures. This allows cells to grow in a more natural environment and enables a more realistic mimicry of cellular interactions (6).

Spheroid formation is of critical interest, particularly in cancer research, as spheroids can better mimic the behavior and characteristics of cancer cells within tumor tissue. These structures can provide features, such as self-renewal at the single-cell level, differentiation ability, and the modeling of cellular interactions within the tumor microenvironment (7).

Sucrose, a disaccharide composed of simple sugars, is a naturally occurring sweetener. Recent studies suggest that sucrose may be associated with cancer development and can promote the growth of cancer cells (8). Of note, cell culture studies have provided crucial information in this regard. In the study by Goncalves *et al* (9), it was found that high fructose increased intestinal tumor growth and cell proliferation in mice, and it increased the risk of metastasis. Similarly, other studies have also indicated that sucrose may have similar effects in breast cancer, prostate cancer and a number of other types of cancer (10-13) . It is reported that sucrose can regulate the activity of enzymes within the cell and promote spheroid formation. Although there have been studies on the effects of sucrose on spheroid formation, further research is required to provide a clear answer on whether sucrose supports spheroid formation (14-16).

Alpha-fetoprotein (AFP) is a protein produced in the liver and yolk sac of a developing fetus (17). AFP is used as a biomarker for the early detection and diagnosis of HCC, particularly in patients with chronic hepatitis B or C infection, and cirrhosis. Elevated AFP levels can indicate HCC; however, it is not specific to HCC and can also rise in other conditions, such as liver regeneration or certain gastrointestinal tumors (18,19). AFP levels can also provide information about the prognosis of HCC. High AFP levels are generally associated with more advanced disease and a poor prognosis (17). Albumin, synthesized by the liver, is the main protein found in the blood (21). In liver diseases, including HCC, the ability of the liver to produce albumin is often impaired (22). Low albumin levels are associated with a poor prognosis in HCC and can reflect the severity of underlying liver dysfunction. Albumin levels can also provide information about the nutritional status of the patient, which is crucial for the management of HCC, as malnutrition can affect the ability of the patient to tolerate treatment and recover (23).

While AFP is used for the detection, monitoring and prognosis of HCC, albumin levels provide information about the functional status of the liver, as well as the overall prognosis and nutritional status of the patient in the context of HCC. Both markers are crucial for the management of patients with liver cancer.

Therefore, the present study investigated the effects of sucrose on spheroid formation, AFP levels and albumin secretion, as well as on the morphology of living cancer cells. The term ‘living cancer cells’ refers to the HepG2 cell line (ATCC HB-8065), not primary tumor-derived cells.

## Materials and methods

### Cells and cell culture

The human liver cancer cell line, HepG2, (American Type Culture Collection^®^, cat. no. HB-8065) was incubated at 37˚C in a 5% CO_2_ environment in Dulbecco's modified Eagle's medium (DMEM, LM-T1720/100, Biosera) supplemented with 10% (v/v) heat-inactivated fetal bovine serum and 1% antibiotics (10 mg/ml streptomycin and 10,000 U/ml penicillin, PAN-Biotech^®^ GmbH). Sucrose (Thermo Fisher Scientific, Inc.) was dissolved in distilled water at a concentration of 10 mmol/l. The experiment was conducted in two different cell culture environments. The first environment was a conventional two-dimensional (2D) cell culture, while the second was a three-dimensional (3D) cell culture environment created using Geltrex (Gibco™; Thermo Fisher Scientific, Inc.), a hydrogel composed primarily of extracellular matrix proteins such as laminin, collagen type IV and heparan sulfate proteoglycans. On the day of the experiment, sucrose was prepared at the required concentrations. The groups treated with sucrose included HepG2 cells grown in the 2D culture and HepG2 cells grown on Geltrex. Sucrose was not applied to the control groups. For spheroid generation from HepG2 cells, the method developed by Tok *et al* (24) was used. A 10 mM sucrose stock solution was prepared in sterile distilled water and stored at 4˚C. For all the experiments, cells were treated with sucrose at a final working concentration of 10 mM by the direct addition of the stock solution to the culture medium. All experiments were performed with n=3 independent biological replicates, unless otherwise stated.

### Live cell imaging

HepG2 cells were cultured under 2D and 3D conditions. For 3D culture, spheroids were generated using Geltrex-coated plates as described above. Sucrose was prepared as a 10 mM stock solution and applied to the experimental groups at a final concentration of 10 mM, while the control groups received no sucrose treatment. Live cell membrane and nuclear staining were performed using the Image-iT™ LIVE Plasma Membrane and Nuclear Labeling kit (cat. no. I34406, Invitrogen; Thermo Fisher Scientific, Inc.) according to the manufacturer's instructions to assess cell viability and membrane integrity. Images were acquired using a Zeiss LSM 700 confocal microscope (Carl Zeiss AG) fluorescence/confocal microscope under identical imaging settings for all experimental groups.

### Histochemistry

Both the 2D and 3D cell culture environments were evaluated using periodic acid-Schiff (PAS; MilliporeSigma) staining. For this purpose, the 2D and 3D cell cultures were incubated for 7 days at 37˚C in a 5% CO_2_ environment. Following fixation with formaldehyde (Merck KGaA) at room temperature for 15 min, the histological staining temperature and duration have been specified as room temperature for 30 min. was performed. PAS is a staining technique used to detect water-insoluble polysaccharides in tissues. The reaction between periodic acid and adjacent diols in sugars forms a pair of aldehydes. These aldehydes then react with Schiff's reagent, producing a purple-magenta color. The presence of glycogen deposits within the cells growing 2D or forming spheroids was examined using high-resolution light microscopy as Zeiss Primovert (Carl Zeiss AG).

PAS staining was performed on all experimental groups. The changes in glycogen deposits due to the effect of sucrose were thus examined. Additionally, ImageJ software (version 1.53k, National Institutes of Health) was used to clearly identify the regions stained with magenta by the PAS stain.

### Morphological analysis of the membrane integrity of living cancer cells

Cells from each group were labeled using plasma membrane labeling kit for live cells (Image-IT™ LIVE Plasma Membrane and Nuclear Labeling Kit; Invitrogen; Thermo Fisher Scientific, Inc.). All experimental groups were labeled with a live membrane marker using the Image-iT™ LIVE Plasma Membrane and Nuclear Labeling kit (cat. no. I34406, Invitrogen; Thermo Fisher Scientific, Inc.) at room temperature according to the manufacturer's instructions. and imaged with a confocal microscope as Zeiss LSM 700 confocal microscope (Carl Zeiss, Germany). The changes in the cell membrane in groups treated with sucrose were statistically compared to the control groups.

### Immunofluorescence staining

For the immunofluorescent labeling of spheroids in 3D cell culture and the cells growing in 2D culture, whole-mount immunofluorescence staining was performed as previously described by Tok *et al* (24). Both spheroids and 2D cells were first fixed with 4% formaldehyde at room temperature for 37˚C and 30-15 min, respectively, then washed three times with CellO™-IF (Cellorama). wash buffer. Primary antibodies against AFP (cat. no. MA5-29006, Thermo Fisher Scientific, Inc.; 1:200 dilution) and albumin (cat. no. A3293, MilliporeSigma). (1:50 dilution) were incubated at 37˚C with the cells for 1 h. Following incubation, the cells were washed three times with CellO™-IF wash buffer. Subsequently, AlexaFluor 488 (cat. no. A-11008, Thermo Fisher Scientific, Inc.) and AlexaFluor 550 (cat. no. 84541, Thermo Fisher Scientific, Inc.) conjugated secondary antibodies (dilution 1:500) were added and incubated at room temperature for 1 h in the dark to prevent photobleaching. This step was followed by three additional washes with phosphate-buffered saline (PBS; cat. no. P4417, MilliporeSigma) to remove any excess secondary antibodies. All steps were performed for 1 h at 37˚C. Following staining, spheroids and 2D growing cells were mounted with a mounting medium consisting of glycerol (cat. no. G6279, MilliporeSigma), PBS and DAPI (cat. no. D1306, Thermo Fisher Scientific, Inc.) (1 µg/ml) to stain the nuclei. The samples were then imaged using a Zeiss LSM 700 laser scanning confocal microscope (Carl Zeiss AG) at appropriate excitation/emission wavelengths for AlexaFluor dyes and DAPI. Image analysis, including the localization of AFP and albumin, was performed using ZEN Black software (version 2012, Zeiss AG). and ImageJ software (version 1.53t; National Institutes of Health). Images were acquired using a Zeiss LSM 700 confocal microscope (Carl Zeiss AG) with excitation/emission wavelengths of 488/525 nm for FITC and 561/595 nm for Texas Red [this refers to a fluorescence detection channel (filter) of the Zeiss LSM 700 confocal microscope] under identical imaging settings for all experimental groups. Plasma membrane integrity and cell viability were compared between the different experimental groups. The localization of AFP and albumin, as well as the live cells and their plasma membrane were comparatively analyzed using Zen image analysis software and Image J software.

### Proliferative changes in liver cancer cells growing 2D- and 3D cultures

DAPI-stained nuclei were quantified using ImageJ software (version 1.53t; National Institutes of Health). Cell counting was performed in five randomly selected, non-overlapping fields per sample at x20 magnification, each representing a defined and constant field area. All images were acquired under identical exposure and imaging conditions, and the mean cell number per field was used for statistical analysis.

### Statistical analysis

All experiments were performed using three independent biological replicates (n=3). Quantitative data are presented as the mean ± standard deviation (SD). Statistical analyses were conducted using GraphPad Prism version 8.01 (Dotmatics). The experimental design included two independent factors: culture condition (2D vs. 3D) and treatment (control vs. sucrose). Accordingly, data were analyzed using two-way analysis of variance (ANOVA) to evaluate the main effects of culture condition and treatment, as well as their interactions. When significant main effects or interactions were detected, Tukey's multiple comparisons test was applied for post hoc analysis. A P-value <0.001 was considered to indicate a statistically significant difference.

## Results

### Sucrose enhances the proliferation and spheroid formation of HepG2 cells

**Figure 1:**
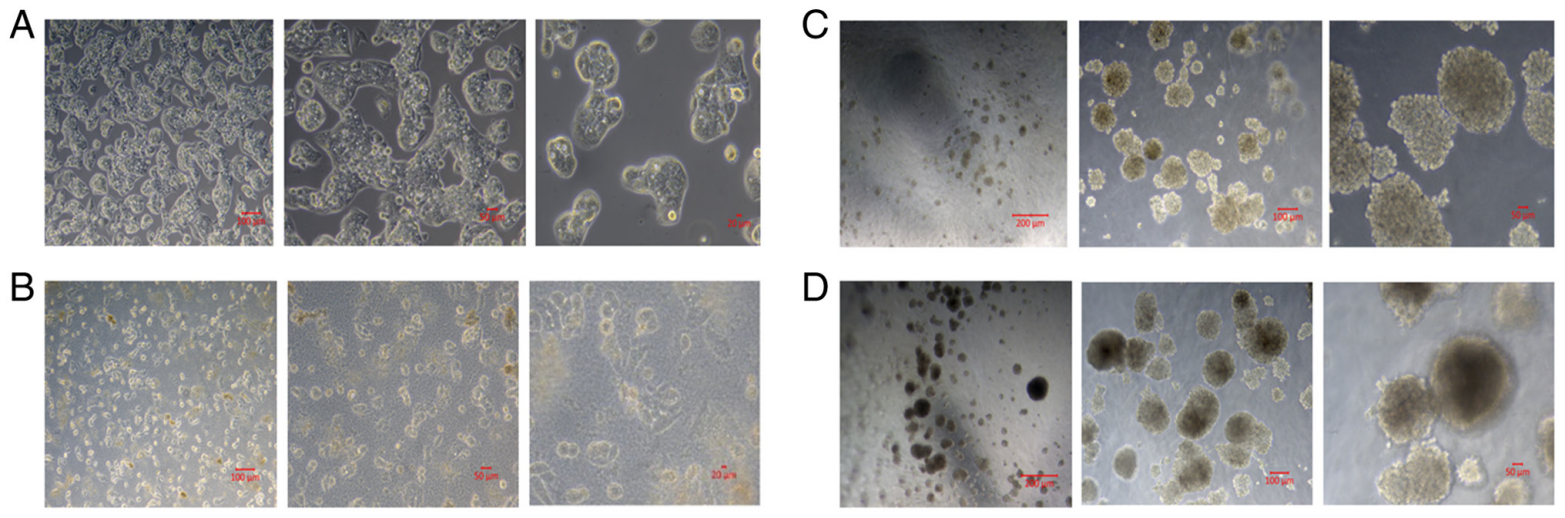
Representative live images of experimental groups at day 7. Data are presented as mean ± SD (n=3). (A) 2D-cultured HepG2 cells under standard culture conditions, (B) 2D-cultured HepG2 cells treated with 10 mM sucrose, (C) 3D-cultured HepG2 spheroids, and (D) 3D-cultured HepG2 spheroids treated with 10 mM sucrose.

The HepG2 cells were examined and imaged every other day under a phase contrast microscope. This allowed for a visual representation of the changes at the morphology of living cancer cells in addition to proliferation rate. In the 2D growing experimental group, the cell number increased significantly following sucrose exposure, as evidenced by the images. In the 3D cell culture environments created with Geltrex, spheroid formation was evident in both groups, treated and untreated spheroids with sucrose. However, the numbers and size of the spheroids were notably higher following treatment with sucrose. Live-cell imaging was performed at day 7 as the experimental endpoint to compare morphological differences between the experimental groups (Fig. 1).

### Sucrose affects PAS-positive carbohydrate accumulation in 2D- and 3D-growing HepG2 cells

**Figure 2:**
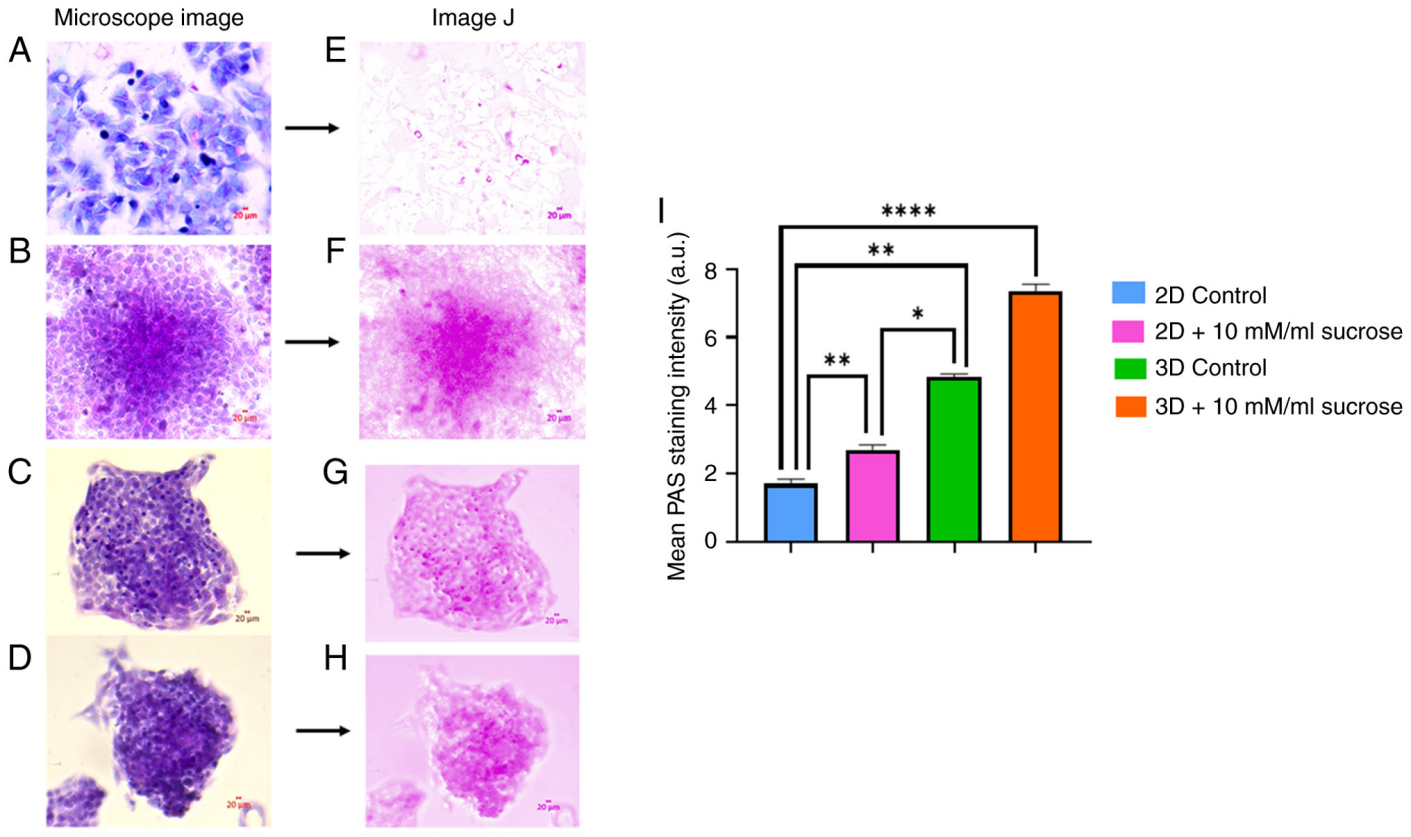
PAS-stained (A) 2D-growing HepG2 cells under traditional culture conditions, (B) 2D-growing HepG2 cells following treatment with 10 mM sucrose, (C) 3D-growing HepG2 spheroids, and (D) 3D-growing HepG2 spheroids after treatment with 10 mM sucrose. Panels (E-H) demonstrate the visualization of PAS staining using ImageJ software. (I) Quantification of PAS staining intensity. Data are presented as mean ± SD (n=3). Statistical analysis was performed using two-way ANOVA to evaluate the effects of culture condition (2D vs. 3D), treatment (control vs. sucrose), and their interaction, followed by Tukey's multiple comparisons test. A P-value <0.001 was considered to indicate a statistically significant difference. ^*^P=0.0523; ^**^P=0.0079 and ^****^P<0.0001.

PAS staining revealed an increase in PAS-positive intracellular material, which is commonly associated with glycogen-rich content. However, as amylase digestion control was not performed, these results should be interpreted as indicative of PAS-positive carbohydrate accumulation rather than definitive glycogen identification (Fig. 2A-H); the quantitative analysis of PAS-positive areas, performed using GraphPad Prism software, demonstrated a significant increase in sucrose-treated groups compared to the controls (Fig. 2I).

### Measurement of proliferative changes in liver cancer cells growing in 2D and 3D culture

**Figure 3:**
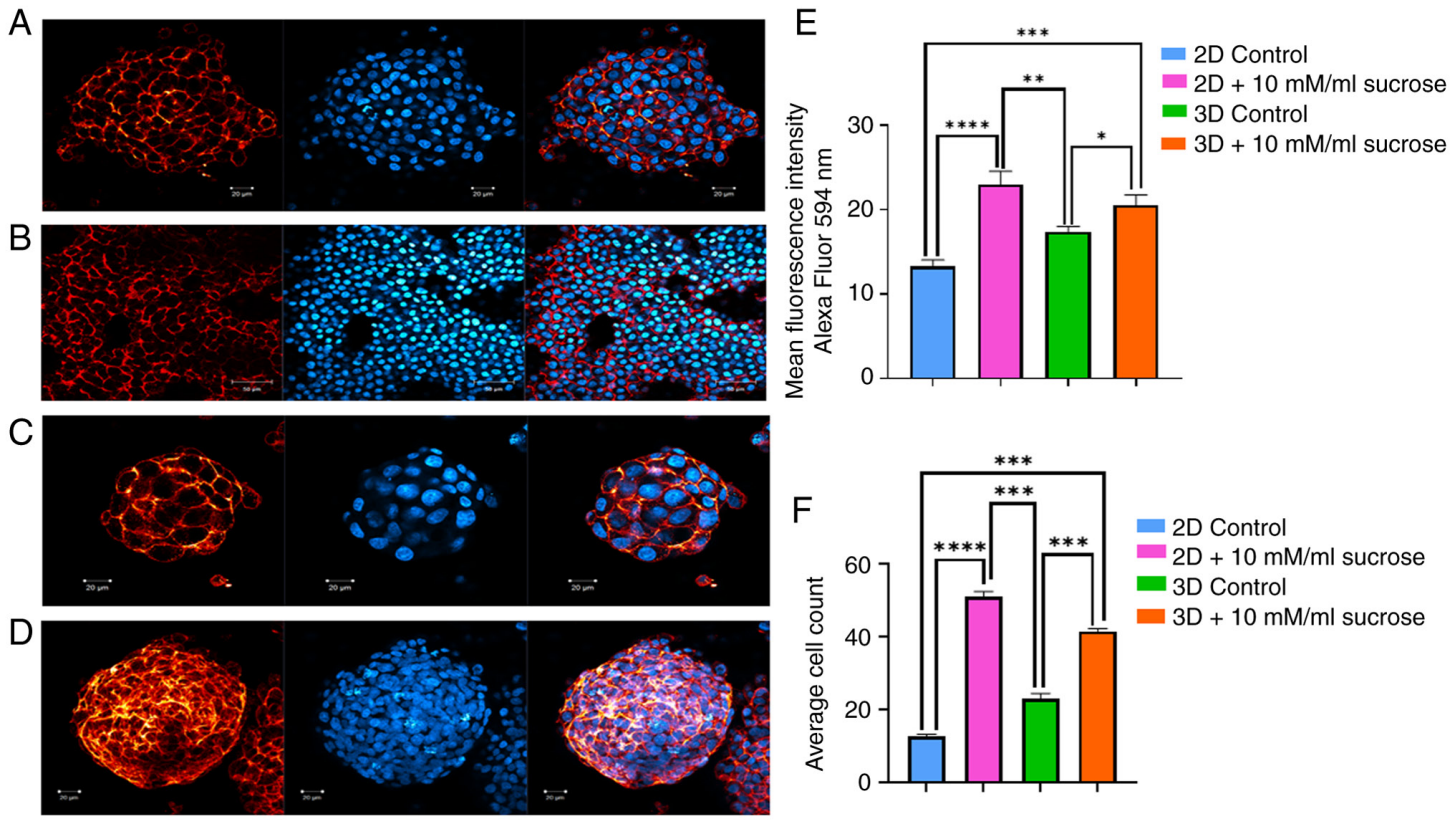
Live-membrane and DAPI-stained (A) 2D-growing HepG2 cells under traditional culture conditions, (B) 2D-growing HepG2 cells following treatment with 10 mM sucrose, (C) 3D-growing HepG2 spheroids, and (D) 3D-growing HepG2 spheroids following treatment with 10 mM sucrose. (E) Quantification of membrane integrity. (F) Average cell count graph. (A-D) The image on the left panel illustrates the plasma membrane, the image in the middle panel illustrates the cell nucleus, and the image on the right panel represents the merged image. Data are presented as the mean ± SD (n=3). Statistical analysis was performed using two-way ANOVA to evaluate the effects of culture condition, treatment, and their interaction, followed by Tukey's multiple comparisons test. A P-value <0.001 was considered to indicate a statistically significant difference, as follows: (E) ^*^P<0.226; ^**^P<0.255; ^***^P<0.005 and ^****^P<0.0001; (F) ^***^P<0.0002 and ^****^P<0.0001.

For each group, changes in cell proliferation in response to sucrose treatment were investigated by counting the nuclei of cells stained with DAPI using ImageJ software. Representative confocal images of DAPI-stained cells from 2D cultures are presented in Fig. 3A and B, while images from 3D cultures are presented in Fig. 3C and D. In these images, cell nuclei appear as blue fluorescent structures located in the central regions of the images due to DAPI staining. An increase in cell proliferation was observed under sucrose treatment. The quantitative analysis of nuclei counts is presented in Fig. 3F. Overall, an increase in proliferation was detected under the influence of sucrose.

### Sucrose alters HepG2 cell behavior without affecting membrane integrity

HepG2 cells were labeled with live cell membrane stain. There was no significant difference in the staining of cell membranes in the experimental groups. However, the results of this experiment also confirmed the considerable effect of sucrose on the proliferation and spheroid formation of HepG2 cells. The sucrose-treated groups exhibited increased cell numbers and bigger, more spheroids, further validating the impact of sucrose.

### Morphology of liver cancer cells does not significantly change over time under the effects of sucrose

As illustrated in Fig. 3, live cell membrane staining confirmed the presence of healthy living cancer cells with precisely labeled cell membranes and nuclei in all experimental groups. Notably, sucrose treatment did not markedly alter membrane integrity.

### AFP and albumin expression

**Figure 4:**
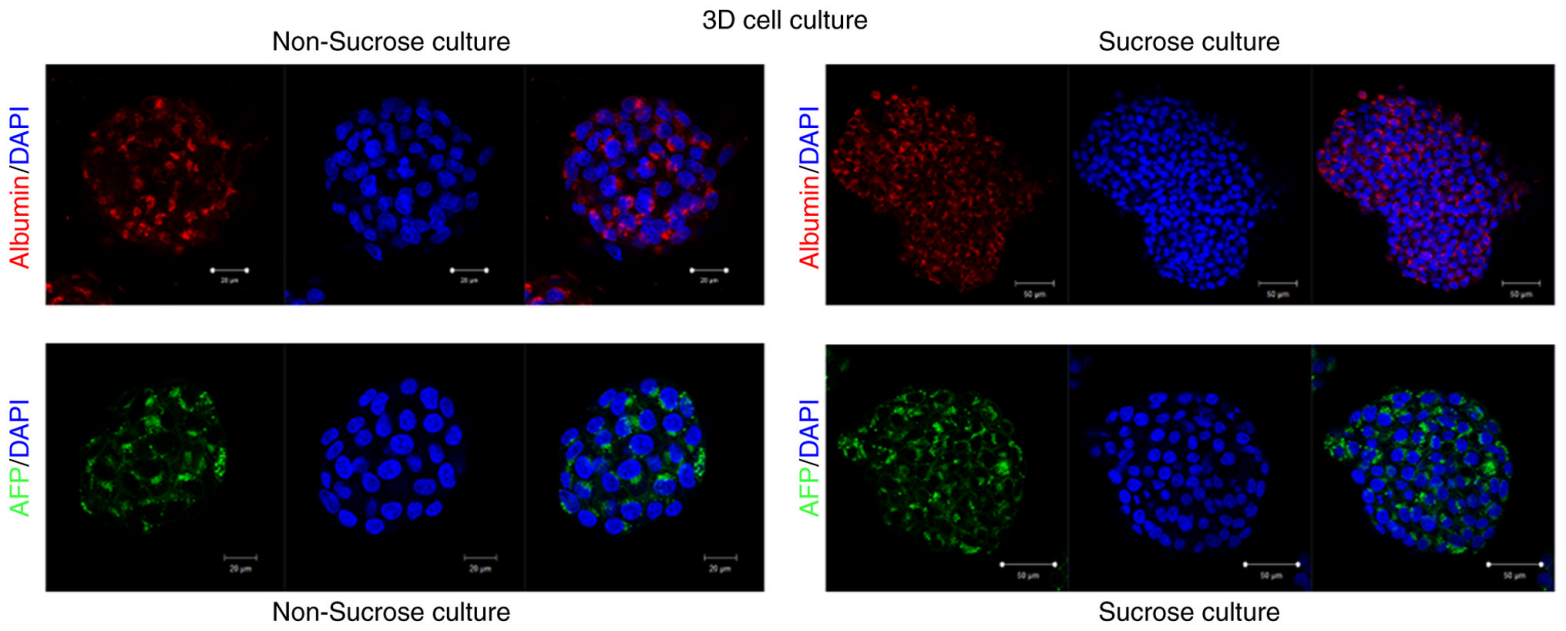
Representative confocal immunofluorescence images of AFP and albumin expression in HepG2 cells cultured under two-dimensional (2D) and three-dimensional (3D) conditions. (A) 2D-cultured HepG2 cells under standard culture conditions and 2D-cultured HepG2 cells treated with 10 mM sucrose. (B) 3D-cultured HepG2 spheroids and 3D-cultured HepG2 spheroids treated with 10 mM sucrose. AFP is shown in green and albumin in red. Images were acquired using confocal microscopy under identical imaging conditions. (A-D) The image on the left panel illustrates the antibody staining (AFP or albumin), the image in the middle panel illustrates the cell nucleus, and the image on the right panel represents the merged image. Data are presented as the mean ± SD (n=3). AFP, alpha-fetoprotein.

The localization of AFP and albumin in the experimental groups labeled with monoclonal antibodies, as well as the changes in these antibodies due to the effect of sucrose, are demonstrated in Fig. 4. Fig. 4 presents representative confocal microscopy images illustrating AFP and albumin expression patterns under control and sucrose-treated conditions. Confocal imaging revealed that sucrose exposure led to significant changes in the secretion dynamics of both markers. Analyses using confocal microscopy at the secretion of AFP and albumin confirmed markedly meaningful changes under the effect of sucrose. The expression of AFP in the cytosol of liver cancer cells increased following sucrose exposure, whereas no marked change was observed in albumin expression.

## Discussion

The present study investigated how sucrose affects liver cancer cells (HepG2) in both 2D and 3D environments. Sucrose significantly increased the growth rate of HepG2 cells in 2D cultures and promoted their ability to form spheroids in 3D culture, potentially enhancing tumor aggressiveness. Live-cell imaging in the present study was performed at the experimental endpoint (day 7). Therefore, the temporal dynamics of sucrose-induced effects during the early stages of spheroid formation were not addressed in the present study. Future studies incorporating multiple time points would allow for a more comprehensive evaluation of these effects. Sucrose treatment has been shown to be associated with an increased PAS-positive intracellular carbohydrate accumulation in both 2D and 3D cancer cell models (22,23). Herein, live cell imaging revealed healthy cell membranes and nuclei even following sucrose treatment, suggesting internal metabolic changes may be driving cancer cell growth. When the expression profiles of AFP and albumin were considered together, the findings suggested that sucrose may be associated with changes related to a malignant phenotype. The observed increase in AFP expression, a commonly used cancer marker, supports this possibility. However, the observed albumin expression in the cells suggests that they may not have fully lost their differentiation characteristics, indicating that the effects could reflect an early or partial malignant response rather than a fully established malignant transformation (24,25).

While the present study focused on liver cancer cells *in vitro*, existing literature suggests an association between high dietary sugar intake and cancer-related outcomes across various experimental and epidemiological models. Previous preclinical studies have reported that high-sucrose or high-fructose diets are accompanied by alterations in cellular metabolic and inflammatory processes; however, these observations are derived from systemic models and were not directly assessed in the present study. In humans and primates, additional sugar consumption has been extensively investigated in relation to metabolic syndrome, a known risk factor for cancer, despite the limited number of prospective studies directly linking sugar intake to cancer incidence (25). Notably, a higher sugar consumption following a breast cancer diagnosis has been shown to be associated with increased all-cause and cancer-specific mortality (25).

It should be noted that the observed effects of sucrose treatment may, at least in part, be influenced by changes in medium osmolarity. Previous research has demonstrated that alterations in extracellular osmolarity can modulate cancer cell behavior, including proliferation, metabolism and spheroid formation, independent of the specific solute used (26). In the present study, an osmolarity-matched inert control, such as mannitol, was not included. Therefore, it cannot be fully excluded that some of the observed cellular responses may be attributable to osmotic stress rather than sucrose-specific metabolic effects (27,28). This limitation should be considered when interpreting the results, and future studies incorporating appropriate osmolarity controls will be necessary to distinguish sucrose-specific effects from general osmotic responses.

In comparison to starch-based control diets, data from three breast carcinoma models demonstrated that sucrose-enriched diets not only accelerated the growth and initiation of mammary gland tumors, but also significantly increased the lung metastasis potential of mammary carcinoma (29). In total, 30% of 6-month-old MMTV/neu mice fed a starch control diet had detectable tumors; however, in animals fed a sucrose-enriched diet (125, 250, and 500 g/kg, respectively), 50% of the mice developed mammary tumors. The average tumor weight of the mice fed a 250 g/kg sucrose diet was 50 mg greater than that of the starch control group, suggesting that sucrose both accelerated the growth of mammary cancer cells and delayed the start of breast tumors (29).

In addition, in a previous study, a positive association has been found between the consumption of soft drinks and the risk of developing HCC, which was present in continuous analyses and for the highest categories of intake in the cohort, but also in the nested case-control subset after adjustment for hepatitis status and liver function score (13). In comparison to the control group (13.1±1.6), there were considerably more tumors located in the proximal intestine (21.9±1.4) among those who consumed high-sucrose diets (13). The mean weight of primary tumors and the total number of lung metastases in the 4T1 cell-bearing animals were statistically substantially greater in the 250 g/kg sucrose diet group compared to the starch control diet group (29). As shown in several clinical or preclinical studies, sucrose or sucrose supplemented nutriments could cause foodborne precancerous reactions at the molecular level (13,29).

The present study aimed to demonstrate the effects of sucrose on liver cancer cells in *in vitro* 2D- and 3D-cell culture models. These findings, together with existing research, suggest that dietary sugar may influence liver cancer-related cellular behaviors under *in vitro* conditions. The analysis of protein markers revealed a concerning trend as reported in the previous clinical and preclinical studies: Sucrose increased AFP (a cancer marker) levels, while the levels of albumin (a marker for healthy liver function) remained unaltered, potentially indicating a shift towards a more malignant state. A key limitation of the present study is the absence of pathway-level or molecular analyses to explain the observed changes in cell growth and AFP expression following sucrose exposure. No metabolic, signaling, or transcriptional pathway-specific experiments were performed. Therefore, the findings of the present study should be interpreted as phenotypic observations rather than evidence of a defined molecular mechanism. Future studies incorporating targeted pathway analyses will be necessary to elucidate the underlying biological processes.

In conclusion, the present study represents an initial investigation into the effects of sucrose exposure on liver cancer cells cultured in both two-dimensional and three-dimensional *in vitro* models. By analyzing whole samples within their native 2D or 3D microenvironments, this approach provides an integrated view of cellular growth and spheroid organization under controlled conditions. Our results show that sucrose exposure at 10 mM is associated with increased cell proliferation and enhanced spheroid formation compared to untreated controls. While sucrose has traditionally been used as a supportive component in cell culture systems, these findings indicate that it may modulate specific cancer cell-related behaviors, particularly growth rate and three-dimensional organization, rather than directly promoting cancer aggressiveness. Notably, the observed effects are limited to *in vitro* models and do not address invasive, metastatic, or *in vivo* tumor behavior. From a methodological perspective, controlled enhancement of spheroid formation may contribute to the development of more reproducible 3D liver cancer models for drug screening applications. At the same time, these findings highlight the need for careful optimization of cell culture conditions to avoid unintended alterations in cellular phenotypes. Further studies incorporating osmolarity-matched controls and direct metabolic analyses will be necessary to clarify the biological mechanisms underlying these observations.

## Data Availability

The data generated in the present study may be requested from the corresponding author.
